# Sensory Nerve Regulation via H3K27 Demethylation Revealed in Akermanite Composite Microspheres Repairing Maxillofacial Bone Defect

**DOI:** 10.1002/advs.202400242

**Published:** 2024-06-14

**Authors:** Kaijun Gu, Yu Tan, Sitong Li, Siyue Chen, Kaili Lin, Yanmei Tang, Min Zhu

**Affiliations:** ^1^ Center of Craniofacial Orthodontics, Department of Oral and Cranio‐Maxillofacial Surgery, Shanghai Ninth People's Hospital, Shanghai Jiao Tong University School of Medicine, College of Stomatology, Shanghai Jiao Tong University, National Center for Stomatology, National Clinical Research Center for Oral Diseases, Shanghai Key Laboratory of Stomatology Shanghai Research Institute of Stomatology Shanghai 200011 China; ^2^ Department of Orthodontics, Shanghai Stomatological Hospital and School of Stomatology Fudan University Shanghai Key Laboratory of Craniomaxillofacial Development and Diseases, Fudan University Shanghai 200001 China; ^3^ Department of Orthodontics Shanghai Ninth People’s Hospital affiliated to Shanghai Jiao Tong University School of Medicine Shanghai 200011 China

**Keywords:** bioactive ions, bone regeneration, composite microspheres, methylation modification, sensory nerve

## Abstract

Maxillofacial bone defects exhibit intricate anatomy and irregular morphology, presenting challenges for effective treatment. This study aimed to address these challenges by developing an injectable bioactive composite microsphere, termed D‐P‐Ak (polydopamine‐PLGA‐akermanite), designed to fit within the defect site while minimizing injury. The D‐P‐Ak microspheres biodegraded gradually, releasing calcium, magnesium, and silicon ions, which, notably, not only directly stimulated the osteogenic differentiation of bone marrow mesenchymal stem cells (BMSCs) but also activated sensory nerve cells to secrete calcitonin gene‐related peptide (CGRP), a key factor in bone repair. Moreover, the released CGRP enhanced the osteogenic differentiation of BMSCs through epigenetic methylation modification. Specifically, inhibition of EZH2 and enhancement of KDM6A reduced the trimethylation level of histone 3 at lysine 27 (H3K27), thereby activating the transcription of osteogenic genes such as Runx2 and Osx. The efficacy of the bioactive microspheres in bone repair is validated in a rat mandibular defect model, demonstrating that peripheral nerve response facilitates bone regeneration through epigenetic modification. These findings illuminated a novel strategy for constructing neuroactive osteo‐inductive biomaterials with potential for further clinical applications.

## Graphical abstract

Injectable polydopamine‐PLGA‐Akermanite composite microspheres biodegrades to produce bioactive ions, promoting bone regeneration in irregular maxillofacial defects. Specifically, D‐P‐Ak microspheres activate the trigeminal nerve to release CGRP, synergistically enhancing the osteogenic gene transcription of BMSCs. In detail, CGRP intervenes the KDM6A‐EZH2 balance and causes H3K27 demethylation at an epigenetic level, proving the peripheral nerve regulation in bone repairing.

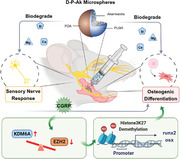

## 1 Introduction

**Figure 1:**
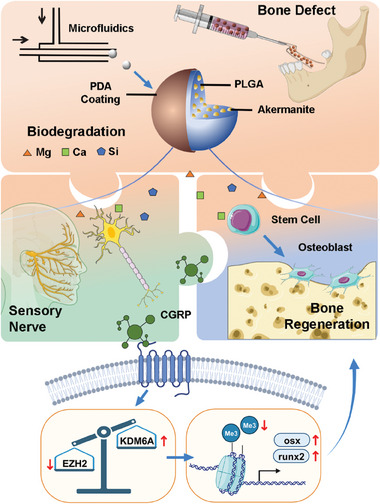
Schematic Diagram of Injectable D‐PLGA‐Ak Composite Microspheres for Maxillofacial Bone Repair. The jigsaw‐like background symbolizes the synergistic effect of osteogenesis promotion by bioactive ions and sensory nerve cells. Uniform‐sized composite microspheres were synthesized using microfluidics, followed by polydopamine coating, and loaded into a syringe for injection into the defect site. As the D‐PLGA‐Ak particles biodegraded, they released bioactive ions crucial for osteo‐differentiation of mesenchymal stem cells. Simultaneously, trigeminal sensory nerve cells responded to these ions and secreted CGRP, initiating the demethylation of histone3 lysine 27, which promotes the transcription of Runx2 and Osx genes.

Maxillofacial bone defects, such as alveolar clefts or teeth extraction sockets, present a unique challenge due to their varied morphology compared to long bone defects. These defects often exhibit irregular shapes and depths, often accompanied by significant soft tissue tension. Consequently, practitioners frequently resort to enlarging incisions to obtain better surgical views during graft procedures, inadvertently causing secondary trauma in treatment. Traditional bone substitutes with prefabricated scaffolds entail longer surgical times and often result in inadequate filling of the defect. In response, there's a growing trend towards the use of injectable bone repair biomaterials such as microspheres and gels. These materials offer significant advantages in minimally invasive repair, providing adequate filling directly at the site and enabling customized restoration of bone loss (**Figure** **1**).

At present, decalcified lyophilized animal bone matrix stands as the most commonly used implantation in bone graft surgery. However, xenogeneic bone matrix lacks osteogenic induction activity and poses a high risk of immunological rejection, often resulting in surgical failure.^[^ ^1^ ^]^ Additionally, without proper degradation rates, bone matrix may persist in the defect for extended periods. Frontline research in bone repair biomaterials is focused on functionalizing bone substitutes through structural design and the incorporation of bioactive substances or seed cells.^[^ ^2^ ^]^ Nevertheless, the application of encapsulated cells or synthesized bioactive molecules is hindered by issues such as instability, immunogenicity, or high cost.^[^ ^3^ ^]^ Hence, there is an urgent need for biodegradable bioactive materials with excellent biosafety and operational performance in clinical practice.

In comparison to traditional integrated scaffolds, microspheres offer advantages such as suitable curved surfaces and limited interacting friction, enhancing mobility during injection and facilitating easier filling of irregular bone defects with minimal invasion. For drug delivery purposes, the inherent spherical structure of microspheres provides a larger specific surface area, thereby enhancing drug‐loading efficiency.^[^ ^4^ ^]^ Akermanite ceramic (Ak), a ternary calcium silicon‐based bioceramic with magnesium, exhibits an appropriate degradation rate, gradually releasing silicon (Si), calcium (Ca), and magnesium (Mg).^[^ ^5^ ^]^ Studies have confirmed that akermanite accelerates bone repair in vivo by promoting angiogenesis and inhibiting RANKL‐induced osteoclast formation.^[^ ^6^ ^]^ However, it's noted that during the initial degradation of akermanite, the surface pH sharply increases, forming an alkaline interface that can affect cell vitality.^[^ ^7^ ^]^ Polymer microspheres, such as poly(lactic acid‐co‐glycolic acid) (PLGA) microspheres, are widely researched as drug delivery systems due to their tunable biodegradability, ease of fabrication, sufficient mechanical properties, and controlled release capability.^[^ ^8^ ^]^ However, limitations of PLGA, such as inadequate osteo‐inductivity and acidic degradation, have led to significant interest in the development of polymer‐ceramic composites by incorporating inorganic ceramic powder into the polymer matrix. This allows for acid‐base neutralization of PLGA‐Ak composites, guiding modification of the interface microenvironment to enhance cell compatibility. By optimizing the ratio of Ak and PLGA, the degradation rate can be adjusted to match the growth rate of new bone tissue.

Advanced functionalization can be achieved through surface modifications, enhancing a biomaterial's ability to integrate with cells and tissues.^[^ ^9^ ^]^ Polydopamine (PDA) coatings derived from biomimetic mussels are believed to promote cell diffusion, proliferation, and migration due to their excellent hydrophilicity and biocompatibility.^[^ ^10^ ^]^ PDA modification enables biomaterials to establish functional surfaces and regulate cell behavior.^[^ ^11^ ^]^

Akermanite produces calcium (Ca), silicon (Si), and magnesium (Mg) ions as degradation byproducts under physiological conditions. In addition to the acknowledged bioactive effects of Ca, Mg, and Si ions in enhancing bone regeneration and mineralization,^[^ ^12^ ^]^ several studies have demonstrated the potential application of Mg in peripheral nerve regeneration.^[^ ^13^ ^]^ The degradation and release of Mg promote the aggregation and release of Calcitonin gene‐related peptide (CGRP).^[^ ^14^ ^]^ CGRP is a crucial neuropeptide that mediates the regulation of stem cells for sensory neurons and acts as a positive regulator in bone regeneration and remodeling, possibly in a concentration‐dependent manner.^[^ ^15^ ^]^ Therefore, biomaterials containing combined ions, including Mg, such as PLGA‐Ak microspheres, may also have neurogenic effects mediated by CGRP, mimicking the neurogenic microenvironment of osteogenesis and exerting synergistic enhancement on bone repair efficacy.

Neuropeptides such as CGRP and substance P are released by sensory nerve cells, serving as messengers between the sensory nerve system and skeletal system to achieve joint regulation of bone repair through various pathways. Among the modulations revealed so far, epigenetic changes caused by histone post‐transcriptional modifications, such as acetylation/deacetylation and methylation/demethylation, play important roles in the epigenetic regulation of bone stem cell functions. For the trimethylation of histone 3 lysine 27 (H3K27me3), enhancer of Zeste Homologous 2 (EZH2), which carries histone methyltransferase activity, increases the level of trimethylation, while lysine demethylase 6A (KDM6A) decreases it. Therefore, the H3K27me3 methylation level is dynamically manipulated by the balance of KDM6A/EZH2. The elevated methylation level is associated with repression of downstream genes, resulting in differences in cellular phenotypes. EZH2 has been shown to downregulate the transcription of cancer suppressor genes and control tumor cell proliferation, migration, and invasive ability by catalyzing H3K27me3.^[^ ^16^ ^]^ In stem cell research, EZH2 leads to Runx2 inhibition and exerts a negative effect on osteogenic differentiation.^[^ ^17^ ^]^ Hence, CGRP is assumed to reduce methylation levels of H3K27me3 and accelerate the transcription of osteogenic genes by balancing KDM6A and EZH2 in this study. In sum, the uncovered CGRP‐initiated epigenetic modulation path for bone regeneration is worthy of further exploration based on previous findings.

With the goal of developing a minimally invasive material that can flexibly adapt to maxillofacial bone defects, we fabricated an injectable D‐PLGA‐Ak microsphere using the microfluidic system, tested its degradation performance and biocompatibility, then explored its osteogenic activity. We focused on the correlation between the material and neurogenic bone regeneration, exploring its mechanism related to histone methylation, and providing a novel strategy for further clinical applications.

## 2 Results

### 2.1 Characterization of (D‐)PLGA‐Ak Microspheres

**Figure 2:**
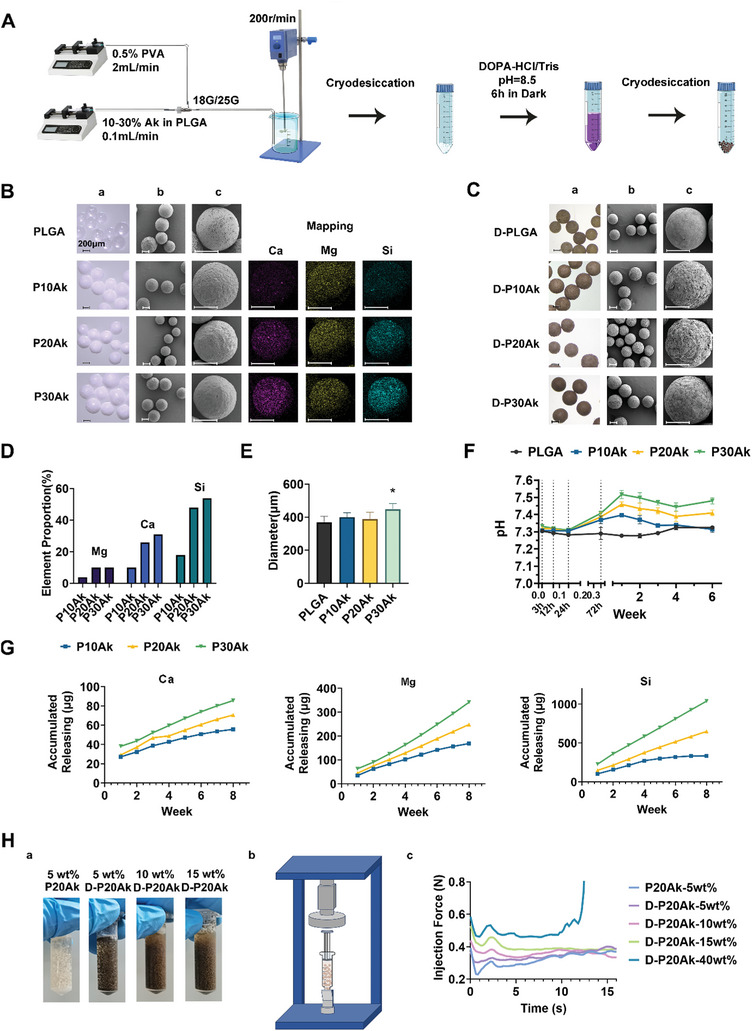
Preparation, Morphological Characteristics and Properties of Microspheres. A) Preparation and coating process of the (D‐)PLGA‐Ak microspheres. B) (a) The stereoscope observation and (b,c) the SEM surface observation of PLGA/P10Ak/P20Ak/P30Ak series microspheres. Mapping of microspheres shows the distribution of Mg/Ca/Si. Scale bar is 200 µm. C) (a) The stereoscope observation and (b,c) the SEM surface observation of D‐PLGA/D‐P10Ak/D‐P20Ak/D‐P30Ak series microspheres with PDA coating. Scale bar is 200 µm. D) Element mapping and analysis of P10Ak/P20Ak/P30Ak series microspheres by energy dispersive spectroscopy analysis. E) Average size of PLGA/P10Ak/P20Ak/P30Ak composite microspheres. 50 microspheres were measured in every group. *P < 0.05. F) pH changes of P‐Ak microspheres in PBS at 37 °C for 6 weeks. 4 mL of the solution were refreshed with fresh PBS every week. 3 parallel samples were tested at every time point. G) Accumulative ion release profiles of P‐Ak composite microspheres in 8 weeks. H) Injectability of the microsphere. (a) microspheres suspended in sheep plasma in gradient weight percentage. (b) microspheres were squeezed through a 1 mL syringe holding to the universal mechanical testing machine. (c) the stress‐time curves of microspheres extruded at a fixed rate of 2 mL min^−1^.

The preparation procedure of (D‐)PLGA‐Ak microspheres was depicted in **Figure** **2**A. Microspheres containing increasing content of akermanite in PLGA (10%−30%wt) were synthesized using a microfluidic platform. Morphology, particle size, and diameter distribution of the (D‐)PLGA‐Ak microspheres were illustrated in Figure 2B–E and Figure S1 (Supporting Information). Among them, P30Ak particles exhibited a larger size, measured at 395 ± 33.1 µm. Pure PLGA microspheres displayed a relatively smooth surface with a diameter of 369.2 ± 35.8 µm, while the PLGA‐Ak microspheres presented rough surfaces due to the incorporation of Ak powders. SEM images of microsphere surfaces revealed pits and embedded powders (Figure 2B). Elemental mapping images showed the presence and homogeneous distribution of Mg, Ca, and Si on the microspheres. Notably, Si comprised the largest proportion of inorganic elements, consistent with the elemental composition of Ak. Additionally, Figure 2C demonstrated that after being coated with PDA, D‐PLGA‐Ak microspheres exhibited a much rougher surface topography, which could promote BMSCs adhesion and proliferation beneath them.^[^ ^18^ ^]^

Another aspect of concern related to the microspheres is the pH change during degradation. The pH changes of PLGA‐Ak microspheres were investigated in vitro by immersion in PBS solution at 37 °C, resulting in the creation of a mild alkaline environment in the PLGA‐Ak group. A steady state was subsequently achieved after 24 hours of immersion in the buffering system. Literature suggests that the pH value of bone reconstructions decreases immediately after implantation in tissue engineering.^[^ ^19^ ^]^ This local acidosis can lead to osteoblast death and subsequent bone loss.^[^ ^20^ ^]^ Providing a base to neutralize endogenous acid production may improve bone mineral accretion. Therefore, the pH increase observed in this research is attractive, yet not detrimental, considering the nature of bone tissue regeneration.

The release behaviors of ions from different PLGA‐Ak microspheres were depicted in Figure 2G, showing continuous accumulation of Ca, Mg, and Si ions over 8 weeks. Higher concentrations and percentages of released Mg, Ca, and Si were detected in P30Ak. Initially, calcium exhibited a burst release. With an increasing load of Ak, the initial burst release and release speed of ions decreased, achieving sustained ion release over a longer period (Figure S1E,F, Supporting Information). The ion release patterns of the D‐PLGA‐Ak microspheres resembled those of the uncoated microspheres but were relatively lower and gentler, indicating that polydopamine coating led to delayed bursts and milder ion release (Figure S1G–I, Supporting Information). The degradation of D‐PLGA‐Ak microspheres in vivo for 8 weeks was displayed in Figure S2 (Supporting Information). The H&E assay of frozen sections showed little inflammation near tissues surrounding injected microspheres.

### 2.2 Injectability of (D‐)PLGA‐Ak Microspheres

At a flow rate of 2 mL mi^−1^n, microspheres ranging from 5wt% to 15wt% in sheep plasma were smoothly extruded through a 1 mL syringe. All particles floated generally in the suspension without piling up at the exit. However, in the 40wt% microspheres group, tight accumulation occurred within the first few seconds of injection, causing the syringe to jam and preventing immediate injection.

### 2.3 Enhanced Biocompatibility, Ccll Proliferation, Adhesion, Migration and Differentiation toward Osteogenesis

**Figure 3:**
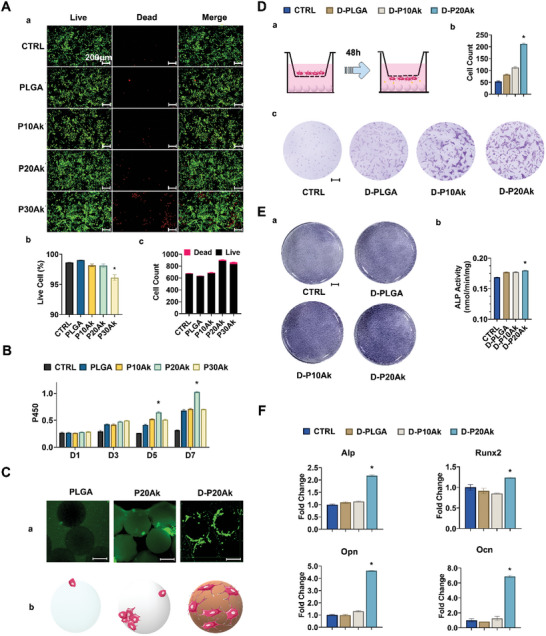
Effect of Microspheres on BMSCs in vitro. A) Biocompatibility of microspheres. (a) Images of Live/Dead analysis for BMSCs showing viability after co‐cultured indirectly with the microspheres’ conditional medium for 2 days. The conditional medium was 7‐day extraction of different microspheres immersing in ɑ‐MEM complete medium respectively. Red staining indicates dead cells and green staining indicates live cells. (b,c) count and proportion of living cells. 3 duplicate samples were set for each group. CTRL, BMSCs cultured with only complete ɑ‐MEM. *P < 0.05, Scale bar is 200 µm. B) CCK‐8 assay of BMSCs indirect co‐cultured with microspheres for 1, 3, 5, and 7 days. CTRL, BMSCs cultured with only complete ɑ‐MEM. 3 duplicate samples were set for each group. *P < 0.05. C) Cell attachment assay of BMSCs implanted on microspheres for 24 hours with (a) calcein staining. (b) sketch map of cell spreading on different surfaces. Scale bar is 200 µm. D) Transwell assay of microspheres. (a) schematic representation of BMSCs migration experiment for 48 hours. (b,c) Images and analysis of migrating cells at the lower surface of the membrane with crystal violet stain. CTRL, both chambers were filled with only complete ɑ‐MEM. 3 visions of view were selected for measurements in each group. Scale bar is 200 µm. *P < 0.05. E) (a) ALP staining images on day 7 and (b) quantitative analysis of ALP activity, the results were normalized with the total protein concentration. CTRL, BMSCs cultured with only complete ɑ‐MEM. 3 duplicate samples were set for each group. Scale bar is 2 mm. *P < 0.05. F) Osteoblastic genes expressions of BMSCs indirectly co‐cultured with microspheres after 4 days. CTRL, BMSCs cultured with only complete ɑ‐MEM. 3 duplicates were tested for each group. The qRT‐PCR results were expressed as the mean ± error. Statistical analysis was performed by one‐way ANOVA. *P < 0.05.

After 48 hours of indirect co‐culture, all viable BMSCs were marked in green, while dead cells were marked in red. Despite the dominance of green signals in **Figure** **3**A, survival analysis revealed relatively weak viability of cells with P30Ak microspheres, suggesting that a high dose of Ak might be unfavorable for BMSC proliferation. Considering the significant difference in size and biocompatibility of P30Ak microspheres, they were excluded from subsequent experiments. Figure 3B demonstrated BMSC proliferation when cultured with PLGA‐Ak microspheres for 7 days, with continuous cell growth observed via CCK‐8 assay for all cases. Higher OD values were observed in the presence of P20Ak microspheres compared to other microspheres on days 5 and 7 of co‐culture.

Strong green fluorescence indicating live BMSCs was observed on all microspheres in Figure 3C, with rarely detected dead cells showing red fluorescence. Confocal laser scanning in Figure S3 (Supporting Information) confirmed the distribution of live BMSCs within the 3D structure, indicating feasibility for cell recruitment and regulation in vivo. Furthermore, cells were observed to attach firmly to the surfaces of D‐PLGA‐Ak microspheres, spreading widely and proliferating vigorously to form microsphere‐cell aggregates after 1 day of incubation. Conversely, cell adhesion was rarely observed on pure PLGA microspheres, and cells attaching to PLGA‐Ak microspheres without polydopamine coating tended to cluster and remain unstretched. Taken together, D‐PLGA‐Ak microspheres were found to be non‐cytotoxic and excellent carriers for supporting cell adhesion and proliferation. Thus, polydopamine‐coated D‐PLGA‐Ak microspheres were considered key objects for subsequent studies.

A Transwell assay was conducted to evaluate whether BMSC migration could be enhanced by ions released from D‐PLGA‐Ak microspheres. In Figure 3D, more stained cells were found in the D‐P20Ak group compared to control and D‐PLGA microspheres. Notably, significant promotion of cell migration was observed for D‐P20Ak microspheres, with migration ratios decreasing in the order of D‐P20Ak > D‐P10Ak > D‐PLGA, corresponding to the amounts of Mg released from these microspheres (refer to Figure 2B). This finding suggested that ion concentration might play a crucial role in homing BMSCs when D‐PLGA‐Ak microspheres were applied in vivo.

Four genes (Alp, Runx2, Opn, Ocn,) and one protein marker (ALP) closely related to the osteogenic differentiation of BMSCs were selected and evaluated alongside the 4 days of indirect co‐culture. At the stage of bone formation and extracellular calcium salt sedimentation, the expression of ALP was high.^[^ ^21^ ^]^ The results in Figure 3E suggested that the ALP activity of the cells cultured with the D‐P20Ak extraction medium was significantly higher than that of the cells cultured on other substrates, indicating the superior effect of D‐P20Ak microspheres in promoting osteogenesis. Furthermore, an ALP staining assay showed semi‐quantitatively higher ALP staining activity in cells cultured with D‐P20Ak extraction medium.

Little significant difference was observed between the control and D‐PLGA group for all genes and markers, suggesting that pure D‐PLGA microspheres had no promoting effect on the osteogenic differentiation of BMSCs. However, osteogenic differentiation of BMSCs was significantly enhanced when cultured with D‐PLGA‐Ak extraction medium, with the D‐P20Ak group demonstrating the strongest ability to upregulate gene expressions. Therefore, it was proposed that D‐P20Ak microspheres would be more efficient in promoting the osteogenic differentiation of BMSCs compared to other D‐PLGA‐Ak microspheres, due to their proper ion loading and long‐term moderate ion release pattern.

### 2.4 D‐PLGA‐Ak Microspheres Promoting Bone Regeneration In Vivo

**Figure 4:**
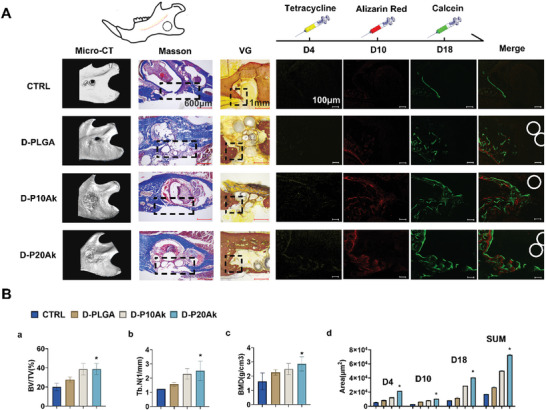
In vivo Bone Repair Potential of D‐P‐Ak Microspheres. A) A schematic demonstration of rat mandibular defect. First column is 3D reconstructed micro‐CT images of mandibles after implantation for 4 weeks. CTRL, the incision was sutured without any filling in the defect. Following column displays histological evaluation of new bone formation revealed by Masson staining and Van Gesson staining. Scale bar is 600 µm and 1 mm respectively. Sequential fluorescent labeling images at 4, 10, 18 days after the operation. 4 duplicate sites were constructed in 4 independent rats for each group of microspheres. Scale bar is 100 µm. B) (a) Quantitative analysis of bone volume/total tissue volume (BV/TV), (b) number of trabecula (Tb. N) and (c) bone mineral density (BMD). (d) Quantitative evaluation of the area of stained bone. Three visions of view were selected for measurements in each group. *P < 0.05.

In **Figure** **4**A, the results of reconstructed micro‐CT 3D models, along with the calculated bone volume/total tissue volume (BV/TV), number of trabeculae, and bone mineral density (BMD) values from in vivo evaluations were presented. Within 4 weeks post‐operation, no evident new bone formation was observed in the unfilled control group, displaying relatively small BV/TV and BMD values. However, with the implantation of D‐PLGA‐Ak microspheres, fragmentary bone tissues were detected sprouting from the defect edge over time, resulting in higher BV/TV and BMD values compared to the control and D‐PLGA group at 4 weeks post‐operation, and significantly higher than those in the other two groups. Histologically, new bone formation and maturity of the newly formed bone tissue were further confirmed by Masson's trichrome staining.

In the control group, defect areas remained vacant with no signs of new bone formation. Conversely, when defects were filled with D‐PLGA‐Ak microspheres, infiltration growth of fibrous tissues was detected, likely due to the space‐occupying and mechanical support effects of the infilled microspheres. In the D‐P20Ak group, a significant amount of collagen‐rich extracellular matrix had filled the spaces between the microspheres, indicating infiltration growth of cells and new bone formation inside the degraded D‐P20Ak microspheres.

Subsequently, sequential fluorescent labeling was performed to evaluate bone formation and mineralization at different stages. The areas stained yellow, red, and green with different calcium‐bonded dyes represented bone regeneration and remodeling at days 4, 10, and 18, respectively. The results showed abundant ossification in all three groups at day 18 (depicted as green). However, compared to the control and D‐PLGA groups, the D‐P20Ak group displayed a larger stained area and enhanced fluorescence intensity. Quantification of the stained bone area indicated promoted bone formation and mineralization in the D‐P20Ak group at each checkpoint of the experiment.

### 2.5 The Regulation of Sensory Nerve Cells on Osteogenesis via CGRP

The intimate relationship between the maxillofacial bone and the neurovascular system, particularly the role of the mandibular neurovascular bundle in nourishing the mandible, has been extensively documented. The peripheral nervous system (PNS) actively engages in osteogenesis by secreting neuropeptides, such as calcitonin gene‐related peptide (CGRP), vasoactive intestinal peptide (VIP), and substance P, among others. These neuropeptides stimulate bone cells, regulating their differentiation and function, thereby contributing to bone generation. Literature also highlighted the vital role of Mg in neurogenic bone regeneration, providing theoretical support for the potential neural activity of D‐P‐Ak microspheres.

**Figure 5:**
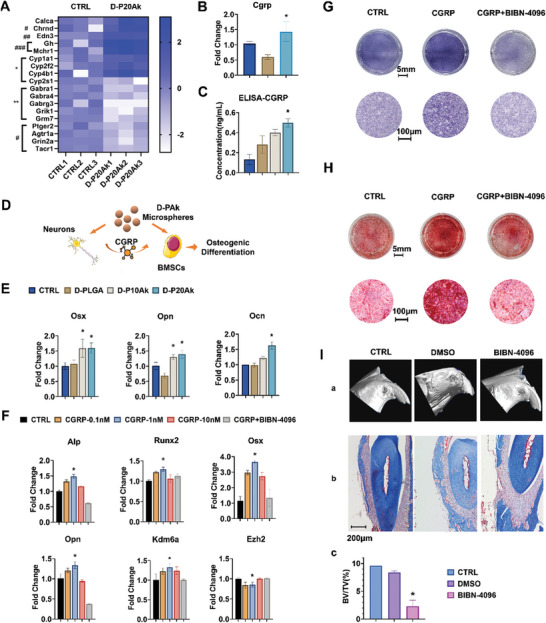
The Synergistic Effect of Trigeminal Sensory Nerve Cells on Osteogenesis. A) Heat map of different expressed genes between trigeminal nerve cells treated with complete F12 medium (CTRL) and D‐P20Ak conditional medium. Fold change data was logarithmically processed. #, genes related to receptors in nerve cells. ##, gene encoding endothelin. ###, genes correlated with neuro‐metabolism. *, genes regarding cytochrome P450 family and lipid metabolism. **, genes encoding central neurotransmitter. Three duplicates were tested for each group, P < 0.05. B) CGRP gene expression of mice trigeminal sensory nerve cells co‐cultured with microsphere extraction medium for three days. CTRL, BMSCs cultured with only complete F12 medium. Three duplicates were tested for each group. *P < 0.05. C) ELISA showing the CGRP concentration in the supernatant of the trigeminal neurons, which were co‐cultured with conditional medium for 3 days. CTRL, BMSCs cultured with only complete F12 medium. 3 duplicates were tested for each group, *P < 0.05. D) Schematic illustration of the experimental verification of synergistic effect and the relationship among “biomaterial, nerve cells and stem cells” triad. E) Osteoblastic genes expressions of BMSCs indirectly co‐cultured with microspheres and nerve cells for 4 days. The conditional medium is 7‐day extraction complete α‐MEM medium collected from trigeminal nerve culture dish. CTRL, BMSCs cultured with only complete ɑ‐MEM. 3 duplicates were tested for each group. The qRT‐PCR results were expressed as the mean ± error. Statistical analysis was performed by one‐way ANOVA. *P < 0.05. F) Osteoblastic genes expressions of BMSCs stimulated by recombinant CGRP protein in gradient concentration for 4 days. CTRL, BMSCs cultured with only complete ɑ‐MEM. 3 duplicates were tested for each group. The qRT‐PCR results were expressed as the mean ± error. Statistical analysis was performed by one‐way ANOVA. *P < 0.05. G) Microscopic images of ALP staining of CGRP‐treated and CGRP+BIBN‐4096‐treated BMSCs on day 4. CTRL, BMSCs cultured with only complete ɑ‐MEM. 3 parallel samples were set for each group. Scale bar is 5 mm and 100 µm respectively. H) Alizarin red staining assays for calcium deposition along with osteo‐inductive culture on day 14. CTRL, BMSCs cultured with only osteo‐inductive medium. 3 parallel samples were set for each group. Scale bar is 5 mm and 100 µm respectively. I) In vivo study of CGRP's role in bone reconstruction. The alveolar bone defects were established at the buccal side of the upper incisor of C57BL/6 mice. BIBN‐4096 were dissolved 750 µg mL^−1^ in DMSO and injected intraperitoneally every 3 days. CTRL, no injection was performed after the surgery. 4 duplicate sites were constructed in 4 independent mice for each group of treatment. (a) 3D reconstructed micro‐CT images of mouse maxillary 4 weeks after operation. (b) Histological evaluation of new alveolar bone formation revealed by Masson staining. (c) Quantitative analysis of BV/TV. Scale bar is 200 µm. 3 samples were scanned and measured in each group of treatment. *P < 0.05.

Thus, this study isolated trigeminal nerve cells from mice and cultured them in vitro with microsphere extraction medium. Peripheral nerve cells co‐cultured with D‐P20Ak microspheres were collected and underwent full transcriptome sequencing. In screening for differentially expressed genes associated with peripheral neural secretion, we noted a significant elevation in the expression of calca, encoding calcitonin gene‐related peptide (CGRP), in trigeminal nerve cells stimulated by D‐P20Ak conditioned medium (**Figure** **5**A). Conversely, other genes implicated in encoding peripheral neurotransmitter and neuropeptide, as reported in the literature, did not show significant differential expression between the control and experimental groups. RT‐PCR results revealed a significant increase in CGRP expression at the mRNA level (Figure 5B), consistent with previously reported neurogenic effects of Mg ions and the sequencing results. Evaluation of the serum level of CGRP protein secreted by trigeminal nerve cells into the extracellular area after microsphere stimulation, using a CGRP‐I ELISA kit, showed a higher concentration in the D‐P20Ak group (Figure 5C), confirming the ability of D‐P20Ak microspheres to elevate CGRP release from trigeminal nerve cells.

Consequently, our forthcoming investigations will focus predominantly on harnessing the neural innervation function within the skeletal system to augment osteogenesis and maintain bone homeostasis. Our primary aim will be to coordinate nerve cells with microspheres to promote bone regeneration through CGRP. We supplemented the culture wells of BMSCs with fresh conditional medium containing a substantial amount of CGRP produced by neurons after microsphere stimulation. This method aimed to closely mimic the environmental interactions observed between microspheres, peripheral nervous cells, and BMSCs in vivo, establishing a mutual and temporally synchronized presence. Within this synergistic culture system of microspheres and nerve cells, BMSCs exhibited a markedly heightened expression of osteogenic genes (Osx, Opn, and Ocn), indicative of a more efficient osteogenic differentiation process (Figure 5E).

Based on the previously detected level of CGRP release, gradient concentrations of CGRP stimulation, ranging from 0.1 nм to 10 nм, and its high‐affinity receptor antagonist olcegepant (BIBN‐4096), were used to incubate BMSCs in vitro, elucidating the role of CGRP in promoting osteogenic differentiation and its lowest optimal effect concentration. Eventually, 1 nм CGRP was determined as the proper concentration for stem cells to differentiate into osteoblasts (see Figure 5F). Additionally, the osteogenic effect of CGRP was validated in vitro with ALP and Alizarin staining assays (see Figure 5G,H) and in vivo via BIBN‐4096 injection in a mouse alveolar defect model (see Figure 5I). Reconstructed micro‐CT 3D images and Masson trichrome staining images illustrated that the alveolar defect was hardly repaired 4 weeks post‐operation with BIBN‐4096 injection. Conversely, normal bone reconstruction in situ was observed in the DMSO control group and sham control group, confirming the crucial role of CGRP binding with its receptor on the surface of stem cells and downstream signaling in the expression of osteogenic genes.

### 2.6 Epigenetic Modification Induced by CGRP Upregulating Osteogenic Gene Transcription

The experimental results indicated that treatment with 1 nм CGRP for 1 hour leads to the overexpression of KDM6A and significant inhibition of EZH2. As a consequence, the trimethylation level of H3K27 is reduced. These effects are largely neutralized by the CGRP‐receptor antagonist BIBN‐4096. Subsequently, the inhibition of H3K27me3 on the transcription of target genes is removed by CGRP stimulation, resulting in the transcription of osteogenic genes such as Runx2 or Osx.

To further validate that CGRP induces changes in histone methylation levels by regulating its downstream histone methyltransferase KDM6A, and thereby promotes osteogenic differentiation, the KDM6A inhibitor GSK‐J4 was applied. GSK‐J4 is an effective dual inhibitor of H3K27me3/me2 demethylases KDM6B and KDM6A, known to promote gene silencing and cell apoptosis by inhibiting KDM6A to regulate histone methylation levels, with applications in cancer treatment.

**Figure 6:**
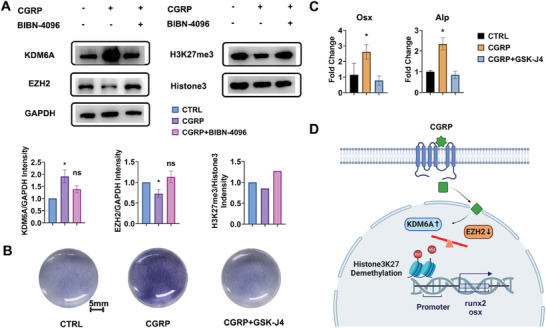
Epigenetic Mechanism of CGRP In Bone Regeneration. A) Western blotting analysis of the expression of methyltransferase of Histone3 in BMSCs treated with 1 nм CGRP or together with nonpeptide CGRP‐receptor antagonist BIBN‐4096 for 1 hour in vitro. Quantitative analysis of the expression of KDM6A, EZH2, H3K27me3 proteins were presented below. CTRL, BMSCs cultured with only complete ɑ‐MEM. 3 parallel samples were analyzed for each group of treatment. *P < 0.05. B) Microscopic images of (a) ALP staining of CGRP‐treated and CGRP+GSK‐J4‐treated BMSCs on day 4. CTRL, BMSCs cultured with only complete ɑ‐MEM. Scale bar is 5 mm. C) Osteoblastic genes expressions of BMSCs stimulated by recombinant CGRP or CGRP+GSK‐J4 for 4 days. CTRL, BMSCs cultured with only complete ɑ‐MEM. 3 duplicates were tested for each group. The qRT‐PCR results were expressed as the mean ± error. Statistical analysis was performed by one‐way ANOVA. *P < 0.05. D) Illustration of the epigenetic modulation initiated by CGRP on osteogenic gene transcription.

The staining results depicted in **Figure** **6**B demonstrate that alkaline phosphatase activity no longer increases after the addition of GSK‐J4 stimulation. Similarly, Figure 6–4 illustrates that the high expression of osteogenic genes induced by CGRP has substantially disappeared under the influence of GSK‐J4. In other words, inhibition of KDM6A weakened the positive effect of upstream CGRP on stem cell osteogenic differentiation. This confirms that KDM6A, as a crucial downstream protein of CGRP, plays a significant role in transducing CGRP stimulation, intervening in osteogenic gene transcription, and influencing cell phenotype.

## 3 Discussion

Defects in facial compartments arising from inflammation, trauma, or tumors often exhibit variations in depth and shape. Many prefabricated scaffolds struggle to meet the demands of such flexible applications. In response to these challenges, we have developed injectable bioactive composite microspheres with uniformly distributed suspension in plasma. D‐P‐Ak microspheres can be smoothly extruded using a 1 mL syringe, possessing the flowability to fully fill defect cavities with minimal trauma, making them suitable candidates for repairing maxillofacial bone defects.

Traditional methods of microsphere preparation, such as spray drying, solvent evaporation, and sol‐gel methods, often result in wide diameter distribution ranges and tight packing during filling, hindering cell, blood vessel, and new bone tissue growth. Conversely, the microfluidic system offers a simple and controllable process to synthesize homogeneous particles, ensuring uniform distribution of microspheres and pores between them. This reduces tight packing during filling and promotes the growth of cells, blood vessels, and new bone tissue.^[^ ^22^ ^]^

Excessive slow degradation is a drawback of decalcified freeze‐dried bone matrix.^[^ ^23^ ^]^ In response, D‐P‐Ak microspheres exhibit significant surface dissolution and cracking at 8 weeks in vivo, consistent with the replacement rate of new tissue repair. Importantly, there is no extreme interface pH or sudden release of certain ions during degradation. The physicochemical properties of the degradation interface remain stable throughout the process, with sustained release of bioactive ions for over 8 weeks, preserving bone inductive bioactivity. These characteristics are vital prerequisites for D‐P‐Ak microspheres as promising bone regeneration materials.

Nerve innervation plays a crucial role in bone regeneration and remodeling, preceding angiogenesis in the early stage.^[^ ^24^ ^]^ CGRP+ nerve fibers are particularly involved in reinnervation after bone injury,^[^ ^25^ ^]^ suggesting an interdependent relationship between bone regeneration and the sensory nerve system mediated by CGRP in bone healing.^[^ ^26^ ^]^ Our data verify that trigeminal sensory nerve cells respond to ions degraded from D‐PLGA‐Ak microspheres by secreting active factors containing CGRP. This outcome aligns with previous findings on the neuroactivity of Mg.^[^ ^27^ ^]^ BMSCs cultured with CGRP administration exhibit lineage differentiation towards osteogenesis, similar to experiments in rats and rabbits.^[^ ^15^, ^28^ ^]^ Disruption of CGRP signaling with CGRP antagonists significantly inhibits new bone formation, as confirmed in this study both in vivo and in vitro.^[^ ^14^ ^]^

Furthermore, CGRP acts as an intermediary regulatory factor for bone repair between BMSCs and other surrounding cellular structures, mediating immune response and controlling the balance of osteogenesis and osteoclasts by promoting IGF‐1 secretion and inhibiting TNF‐α secretion and RANKL‐induced NF‐κB activation.^[^ ^29^ ^]^ CGRP also enhances vessel formation to nourish intramembranous and intrachondral osteogenesis.^[^ ^30^ ^]^ Mechanistically, CGRP activates the cAMP/PKA signaling pathway, inhibiting BMSC apoptosis and promoting BMSC proliferation and osteogenic differentiation by enhancing Wnt/β‐catenin.^[^ ^31^ ^]^ However, the influence of CGRP signals on target gene expression in the nucleus remains largely unexplored.

In recent years, research has shifted towards epigenetic mechanisms, exploring factors initiating MSC lineage differentiation.^[^ ^32^ ^]^ In BMSCs, demethylases such as KDM6A are considered positive regulators of osteogenic differentiation by removing H3K27 trimethylation modifications in the Runx2 promoter regions, initiating the expression of osteogenic‐related genes.^[^ ^17^, ^33^ ^]^ Inhibition of EZH2 enhances osteogenic differentiation by reducing H3K27me3 near transcriptional promoters and enhancing the expression of osteogenic genes.^[^ ^16^, ^17^, ^34^ ^]^ Administration of an Ezh2 inhibitor, hence, was reported to enhance bone formation and prevents bone loss.^[^ ^35^ ^]^ This study validated that CGRP triggers the suppression of EZH2 and upregulation of KDM6A, subsequently reducing the level of H3K27me3 at the promoter, resulting in Runx2 and osx transcription, thus promoting osteogenic differentiation. These findings, together with the association between CGRP and KDM6A/EZH2 balance presented in this research, may provide evidence for mechanism research on downstream pathways of CGRP in bone repair.

This study represented a preliminary exploration into the regulation of bone marrow mesenchymal stem cell differentiation through histone methylation. However, a detailed and comprehensive examination at the chromatin level remains outstanding. The intracellular pathway of CGRP signal transduction and its interaction with histones binding to promoter regions remain elusive. The establishment of a comprehensive theory elucidating the transcriptional regulatory mechanisms of osteogenic genes induced by CGRP is anticipated in future research endeavors.

Acknowledging the complexity of bone defect repair, which involves numerous pathophysiological mechanisms and influencing factors, it is evident that while this study underscores the indispensable role of neural regulation in bone regeneration, the specific repair mechanisms warrant systematic investigation. Overall, there is a discernible dearth of systematic and dynamic research addressing multiple confounding factors. Consequently, neurogenic bone tissue engineering encounters substantial challenges, and achieving synchronous neurogenesis and vascularization in regenerated bone tissue remains elusive. Further studies in this realm are eagerly awaited to propel this promising strategy from experimental stages to clinical application.

## 4 Conclusion

This research develops the injectable PDA‐PLGA‐Akermanite composite microspheres with uniform size, which possess fine injectability and biocompatibility. It stably degrades to produce bioactive ions of Ca, Si, and Mg, and exhibits promotion to BMSCs osteogenic differentiation and bone regeneration both in vitro and in vivo. Sensory nerve cells respond to microsphere stimulation and secrete CGRP, which synergistically promotes bone tissue repair. CGRP, as a key regulatory factor mediating the response, guide stem cells fate through epigenetic pathway of H3K27 methylation modification, achieving neural synergistic function on osteogenesis. This neural synergistic function on osteogenesis offers promising implications for future biomedical applications in maxillofacial bone regeneration.

## 5 Experimental Section

### Materials

Poly(lactic‐co‐glycolic) acid [PLGA; lactide:glycolide = 50:50, carboxyl terminated, Mw (molecular weight) = 110 kDa] were purchased from Sigma. Dichloromethane (DCM) were purchased from Sinopharm Chemical Reagent Co., China. Akermanite powders were synthesized as described in previous publication, using tetraethyl orthosilicate ((C_2_H_5_O)_4_Si, TEOS) in a sol‐gel process, magnesium nitrate hexahydrate (Mg(NO_3_)_2_·6H_2_O) and calcium nitrate tetrahydrate (Ca(NO_3_)_2_·4H_2_O) as raw materials.^[^ ^6b^ ^]^ Briefly, the Ak powder was pressed into tablets and calcined at 1050 °C. After calcining, the ceramic tablets were crushed and sieved using 200 and 400 meshes to obtain Ak particles with diameters of 40–80 µm. Poly(vinyl alcohol) (PVA, Mw = 20–40 kDa, degree of hydrolysis = 97–98.8%) were purchased from Sangon Biotech (Shanghai) Co., Ltd. 3‐Hydroxytyramine hydrochloride (dopamine hydrochloride) was purchased from Sigma‐Aldrich, China. All the chemical reagents were of analytical grade and used as received.

### Preparation of PLGA‐Ak Microspheres with Polydopamine Coating

10% (w/v) PLGA in DCM solution were prepared and stirred 200 r/min for at least 12 hours. 10%/20%/30% (w/v) akermanite were added to the previous solution to establish a gradient concentration. 0.5% PVA (w/v) aqueous solution was heated to 90°C for 2 h.

The microspheres were fabricated by microfluidics using a coaxial needle. The scale of the inner and external needles was 25G and 18G, respectively. The needle and syringes were connected by polytetrafluoroethylene tubes. The dispersed phase (Ak in PLGA/DCM) and the continuous phase (0.5 wt% PVA) were independently pumped into the inlets of the coaxial needle. The inner oil solution was sheared off by the aqueous solution and form the microdroplets out of the device. The flow ratio of the two phases was optimized to obtain spheres of uniform shape and size. Next, the microdroplets were collected in PVA solution and stirred continuously at 200r min^−1^ for at least 2 h in order to evaporate DCM and to stable the microsphere. Then, the microspheres were repeatedly washed with deionized water to remove collecting solution and other additives. Finally, the microspheres were freeze‐dried at −40 °C for 24 h.

To coat with polydopamine, gather microspheres in a tube with 20 mL 2 g L^−1^ polydopamine in 10mм Tris‐HCl solution (pH = 8.5). Put the tube into constant temperature shaker and shaded incubate at 37 °C for at least 6 h. Afterward, the microspheres were repeatedly washed with deionized water and freeze‐dried at −40°C for 24 h.

### Characterizations of PLGA‐Ak Microspheres

*Morphology*: The morphology of PLGA‐Ak core‐shell droplets were examined by a stereomicroscope (Olympus, SZ61). Furthermore, the mean diameters of PLGA‐Ak microspheres were calculated by measuring microspheres randomly in pictures taken by the stereomicroscope. The scanning electron microscopy (SEM, ZEISS GeminiSEM 300) with EDS‐mapping was performed to characterize the surface of all microspheres, polydopamine coated or uncoated. Before observation, the microspheres were subject to gold coating for 40–50s in order to overcome the poor conductivity of the PLGA polymer. The EDS‐mapping showed the proportion of elements on surface layer of the microspheres.

### In vitro Release of Calcium, Magnesium and Silicon ions

The ion release of each group were examined by immersing 30 mg microspheres into 10 mL PBS (pH = 7.4) and incubated at 37 °C for 8 weeks, respectively. The pure PLGA microspheres worked as the control. The release of ions and change pH was measured each week by an inductively coupled plasma optical emission spectrometry (ICPOES, Agilent 730, USA). At each time point proposed, 4 mL of solution was extracted and 4 mL fresh PBS were infused into the system to mimic the dynamics of in vivo metabolism and evaluate the actual concentration of calcium, magnesium and silicon ions released. The accumulated amount of ion at the end of 8 weeks was regarded as the total amount.

### Preparation of Extraction Medium

The extraction medium was prepared similarly to previous solution. 20 mg of microspheres was plated at the bottom of the 6‐well culture plate. The whole uncapped plate was exposed to UV light for 6 h for sterilization. Following manipulations were done inside a biosafety cabinet. Sterile of 8 mL complete medium were added into each well. The culture plate was maintained in an incubator (37 °C, 5% CO_2_, and 95% relative humidity), and the medium was collected after 7 days. The filtration of extraction was immediately performed with Millex‐GP filters (0.22 µm, sterilized, SLGP033RB, Millipore). Thus, 7d extraction medium would provide a basic conditional medium with proper ion concentration for the following in vitro studies.

### Injectability Evaluation

The P20Ak and D‐P20Ak microspheres were weighed and suspended in sheep plasma at a mass ratio of 5%, 10%, 15%, and 40%, respectively. The suspension was extracted with a 1 mL syringe (13G) and the syringe were placed on the universal mechanical testing machine. Using a compression testing module, injection was conducted at a fixed rate of 2 mL min^−1^, and the machine recorded real‐time thrust and displacement every 0.2 seconds. If the microspheres accumulated and could not be pushed forward, the injection would be manually stopped.

### In Vivo Degradation

C57BL/6 mice were purchased from Shanghai Jihui Laboratory Animal Care Co., Ltd. After a week of adaptive breeding, 6‐week‐old mice were randomly divided into 4 groups, as follows: 1) D‐PLGA, 2) D‐P10Ak, 3) D‐P20Ak, 4) D‐P30Ak. Each group has 4 replicates. All mice were anesthetized with 50 mg Kg^−1^ of Zoletil 50 (tiletamine‐zolazepam) (Virbac, France) through intraperitoneal injection. D‐PLGA‐Ak microspheres were injected subcutaneously into the bilateral dorsal pockets of the animals. All groups were euthanized by CO_2_ asphyxia after 8 weeks. The implanted microspheres and neighboring tissues were harvested and fixed with 4% paraformaldehyde (Biosharp, Shanghai, China) for 12 hours.

Half of the samples were pictured using scanning electron microscopy (SEM, ZEISS GeminiSEM 300) to characterize the surface of the polydopamine‐coated microspheres after degradation. The rest were taken to execute frozen section.

### Cell Isolation and Culture

BMSCs were isolated from the bone marrow of 3‐week‐old male Sprague–Dawley (SD) rats. SD rats were purchased from Shanghai Sippr‐BK Laboratory Animal Co.Ltd. (China). Primary BMSCs were cultured in alpha mammal essential medium (α‐MEM) supplemented with 10% (v/v) FBS and 1% (v/v) penicillin‐streptomycin (Gibco, USA). The culture dishes were maintained in an incubator (37 °C, 5% CO_2_, and 95% relative humidity), and the medium was refreshed every third day to remove the nonadherent cells. After reaching 80%–90% confluence, the BMSCs were passaged, and passages 3–4 were used for in vitro experiments in this study.

All mice were purchased from Shanghai Jihui Laboratory Animal Care Co., Ltd. Primary sensory neurons were isolated from the trigeminal ganglion of 7‐day‐old C57BL/6 mice.^[^ ^36^ ^]^ Sharply disconnected from the brain, both ganglia were incubated with iced HBSS without Ca^2+^/Mg^2+^. Chop ganglia into 10–12 pieces using spring scissors. Transfer the tissues into papain solution (0.75 mg in 15 mL HBSS) and 37 °C water bath for 20 min. Centrifuge 200 g for 1 minute. Discard the supernatant and add compound enzyme solution (12 mg Collagenase II+14 mg dispase II in 3 mL HBSS). Incubate in 37 °C water bath for 20 minutes. Centrifuge 400 g for 4 min. Add 0.5 mL prewarmed complete L15 medium (L15 with 5% FCS, penicillin/streptomycin, HEPES, Solarbio Science and Technology Co. Ltd.) Triturate by passing tissue through a 200 mL pipette tip 2–3 times. Make Percoll gradient to separate myelin and nerve debris from trigeminal neurons. Centrifuge at 1300 g for 10 minutes. Remove 4.5 mL from the top, including the area adjacent to the debris. Add 4 mL of complete L15 to the remaining solution in the tube. Centrifuge 1000 g for 6 minutes. The primary sensory neurons were resuspended in preheated 600ul F12 (Corning, CN) complete medium (10% FCS, 1% B27 and 1% penicillin/Streptomycin in F12). Plate cells on laminin/poly‐D‐lysine‐coated coverslips and place plate in an incubator (37 °C, 5% CO_2_, and 95% relative humidity). Two hours after plating, flood wells with 1 mL warm (37 °C) growth medium. Replace medium with fresh warm culture medium every third day. To inhibit the proliferation of non‐neuronal cells, the cells were incubated with 60 µм 5‐Fluoro‐2′‐deoxyuridine (Shanghai Aladdin Biochemical Technology Co., Ltd.). Neurons were used within 5 days of culture.

### Cell Viability Assay

BMSCs were co‐cultured indirectly in a 48‐well plate. Briefly, BMSCs (2×10^4^) were seeded and cultured with the 7‐day extraction of the microspheres as conditional medium for 48 h. The cytotoxicity of PLGA‐Ak microspheres was evaluated using the live/dead staining. After 48 h, cells were incubated with Calcein AM/ propidium iodide (PI) (Beyotime, China) for 30 min and observed under a fluorescence microscope. The influence of microspheres to cell proliferation was assessed by using Cell Counting Kit‐8 (CCK‐8) assays (Beyotime, China). After 1, 3, 5, and 7 days of co‐ culture, the CCK‐8 solution was added to each well and then incubated at 37 °C in 5%CO_2_ for 2 h. Then, the absorbance of the supernatant was read at 490 nm with a microplate reader (Biotek, USA). All the experiments were repeated three times.

### Cell Adhesion

*Laser Scanning Confocal Microscopy*: BMSCs (2.5×10^4^) were seeded at a confocal dish (35 mm in diameter) with 100 sterilized microspheres and place the dish in an incubator (37 °C, 5% CO_2_, and 95% relative humidity). An hour after seeding, flood the dish with 1 mL warm (37 °C) complete medium. After 24 h, cells were incubated with Calcein AM (Beyotime, China) for 30 min. The confocal images were acquired using a laser scanning Leica Stellaris 5 inverted confocal microscope and 3D images were reconstructed to visually demonstrate the cell attachment on the sphere.

### Biological Scanning Electron Microscopy

Take 5 mg of each group of akermanite composite microspheres, lay them in 1.5 mL tubes and sterilize by UV irradiation for 6 hours. Wet the microspheres with sterile PBS solution. BMSCs (1 × 10^6^ mL^−1^) were suspended in complete α‐MEM medium. Add 100 µL suspension to each tube and incubate for an hour. Then, carefully add 1 mL of complete medium. After 24 hours of cultivation, carefully remove the culture medium from the tube, rinse gently once with PBS, and immediately add 1.5 mL of 2.5% glutaraldehyde fixing solution to fix for 12 hours. Pour out the fixing solution, rinse the sample three times with 0.1м, pH 7.0 phosphate buffer for 15 minutes each time, fix the sample with 1% osmium acid solution for 1 hour, carefully remove the osmium acid waste liquid, rinse the sample three times with 0.1м phosphate buffer (pH 7.4) for 15 minutes each time. The sample was dehydrated using an ethanol solution with gradient concentrations (including 30%, 50%, 70%, 80%, 90%, and 95%). Each concentration was treated for 15 minutes, followed by a 20 minute treatment with 100% ethanol. Finally, the sample was placed in fresh 100% ethanol. Dry in a critical point dryer. Use conductive carbon glue to fix the sample on the sample stage, and spray gold with an ion sputtering instrument for about 90 seconds. Eventually, observe under a scanning electron microscope and collect images for analysis.

### Cell Migration

SD rat BMSCs were cocultured with PLGA‐Ak microspheres using a polycarbonate 24‐well Transwell culture chamber with 8 µm pore size membrane (Corning Inc., Corning, NY, USA). Sterilized microspheres were placed at the lower chamber and flooded with 7‐day extraction medium. BMSCs were seeded into the upper chamber and suspended with serum‐free medium. Cells adhered to the upper side of the intersecting membrane was swabbed after 24 h of coculture. Crystal violet staining was performed and the membrane facing lower chamber was observed under an optical microscope.

### Alkaline Phosphate (ALP) Activity and Staining Assay

BMSCs were seeded at a density of 1.2×10^5^ cells per well in 12‐well culture plates in 1.5 mL extraction culture medium, which was replaced every third day. At 4 days of culture, the cells were rinsed with PBS, lysed with 0.1 mL RIPA lysis buffer (EpiZyme, Shanghai, China), and centrifuged at 4 °C, and ALP activity was quantified with an ALP kit (Beyotime, China) according to the manufacturer's protocol. The absorbance was read at 405 nm with a spectrophotometer, and the total protein content was determined using a BCA protein kit (Beyotime, China). The ALP activity was normalized to the total protein concentration.

To intuitively characterize the effect of microspheres on ALP expression, an ALP staining assay was also carried out using a BCIP/NBT Alkaline Phosphatase Color Development Kit (Beyotime, China) at Day 4. Pictures of each well were taken under an optical microscope. All the experiments were repeated three times.

### Alizarin Red Staining Assay

BMSCs were seeded at a density of 1 × 10^5^ cells per well in 12‐well culture plates with 1.5 mL osteogenesis inducing medium (Oricell, MUXMX‐90021). Osteo‐inducing medium were refreshed every three days. The staining were performed on day 14 of culture when calcium nodules were visible through microscope observation. The plate was incubated with alizarin red S solution for 10 min at 37 °C, then washed with PBS for 3 times. Images of each well was captures with an optical microscope. All the experiments were repeated three times.

### Quantitative Real‑time PCR (qRT–PCR)

BMSCs were seeded at a density of 2 × 10^5^ cells per well in 6‐well culture plates in 2.5 mL medium, which was replaced every third day. After being cultured for 4 days, total RNA was isolated from each well using TRIzol Reagent (Invitrogen) and then reverse transcribed to complementary DNA (cDNA) by a PrimeScript RT Kit (TaKaRa, Japan). The expression levels of runt‐related transcription factor‐2 (Runx2), alkaline phosphatase (Alp), osteocalcin (Ocn), osteopontin (Opn), calcitonin gene‐related peptide (Cgrp), and enhancer of zeste homolog 2 (Ezh2), netrin 1 (Ntn1) and nerve growth factor (NGF) were quantified, and glyceraldehyde‐3‐phosphate‐dehydrogenase (Gapdh) was used as a reference gene for normalization. The primer sequences used in this study were presented in additional file.

### Animal Procedures

SD rats of 8‐week‐old were purchased from Shanghai Sippr‐BK Laboratory Animal Co., Ltd. to establish mandibular defects. Subsequent animal experiments were approved by the Institutional Animal Care and Use Committee (IACUC) of Shanghai Ninth People's Hospital Affiliated with Shanghai Jiaotong University, School of Medicine(Approval number: SH9H‐2021‐A16‐1). General anesthesia was achieved via the intraperitoneal injection of 50 mg Kg^−1^ of Zoletil 50 (tiletamine‐zolazepam) (Virbac, France). A 2‐cm horizontal incision was made at the cheek zone of both sides. Blunt separation of masseter muscle were operated to expose the mandibular angle beneath. Defects of 4 mm in diameter were penetrated at the lower back of the mandibular canal vertical to the mandibular nerve vascular path. Each side was identically treated using a drill. To avoid individual differences in the experiments, 32 mandibular defects were constructed in 16 SD rats. Four experimental modalities were randomly assigned to 32 defects (n = 8), as follows: 1) Sham control; 2) D‐PLGA control; 3) D‐P10Ak group; 4) D‐P20Ak group. Microspheres were injected into the defects. At 4, 10, and 18 days after the operation, 16 rats were intraperitoneally injected with tetracycline hydrochloride (TE, 25 mg Kg^−1^), alizarin red (AL, 30 mg Kg^−1^), and calcein (CA, 20 mg Kg^−1^) to trace new bone formation and mineralization. After being implanted for 4 weeks, all groups were euthanized by CO_2_ asphyxia and the specimens (mandibular and scaffolds) were harvested, fixed in 4% paraformaldehyde for 24 h.

In case of evaluating bone regeneration induced by CGRP, 6‐week‐old C57BL/6 mice were purchased from Shanghai Jihui Laboratory Animal Care Co., Ltd. All mice were anesthetized with 50 mg Kg^−1^ of Zoletil 50 (tiletamine‐zolazepam) (Virbac, France) given intraperitoneally. After the separation of gingiva, a circular fenestration defect was constructed bilaterally at the buccal alveolar bone of the maxillary central incisors, 3 mm below the gingival margin. The defect was 2 mm in diameter and reached to the surface of teeth root. The nonpeptide CGRP‐receptor antagonist, olcegepant (BIBN‐4096) (MedChemExpress, China), was dissolved 750 µg mL^−1^ in dimethyl sulfoxide (DMSO). All mice were randomly divided into 3 groups and each group has 4 parallel samples. The experiment group accepted intraperitoneal injection containing 0.9 mg Kg^−1^ olcegepant according to their weight once three days. The DMSO control group was injected with the same volume of diluent with the experiment group at the same frequency, while the control group was left untreated after the operation. After 3 weeks, all groups were euthanized by CO_2_ asphyxia and all the maxillary including incisors were harvested, fixed in 4% paraformaldehyde for 24 h.

### Micro‐CT Analysis

The samples were scanned and imaged using a micro‐CT scanning system (PerkinElemer, QuantumGX, Japan) to evaluate the new bone volume around the defect. The scanning parameters were set at 90 kV, 88 µA and 14 min for scanning time. After scanning, all images with 25 µm voxel size were 3D reconstructed. The ratio of bone volume to total volume (BV/TV), number of trabecular (Tb.N) and bone mineral density (BMD) was calculated using the attached analysis software (Analyze 12.0,Japan).

### Histological and Histomorphometric Analysis

*Paraffin Section*: Half of the samples from all the groups were decalcified with 10% EDTA for 1 month, dehydrated, embedded in paraffin. Mandibular samples were sliced at a thickness of 7 µm and stained using hematoxylin and eosin (H&E) (Solarbio, Beijing, China) and Masson's trichrome staining (Sigma, 1.00485).^[^ ^37^ ^]^

### Frozen Section

After fixation, samples were fused into 30% sucrose solution overnight at 4 °C for dehydration and the solution was replaced with fresh dehydrate 30% sucrose and OCT 1:1 solution for another 24 h. When the tissue was settling to the bottom of the tube, it indicated complete dehydration. The samples were embedded with OCT agent in the embedding box accurately and frozen at −80 °C. 12 µm slices were acquired and attached to anti‐detachment glass slide. Hematoxylin‐eosin staining (H&E) kits were purchased from Solarbio (Beijing, China).

### Hard Tissue Section

Previously injected samples were dehydrated with gradient ethanol and embedded in polymethylmethacrylate (PMMA). Slices with a thickness of 100 µm were cut and polished by a Diamond Band Saw Microtome from an EXAKT cutting‐grinding system (Exakt Company, Germany), and reviewed under the laser scanning confocal microscope (Leica SP8, Germany). Subsequently, the slices were stained with Van Gieson's (VG) staining for histological analysis.^[^ ^38^ ^]^ The stained slices were observed and photographed by an optical microscope.

### Indirect Co‐Culture of Trigeminal Nerve Cells and BMSCs In Vitro

Extraction medium of 7‐day with complete αmem was referred as OsM, while microspheres incubating in F12 complete medium (10% FCS, 1% B27, 1% penicillin/Streptomycin and 60 µм 5‐Fluoro‐2′‐deoxyuridine) for 7 days was referred as NeuM. Primary trigeminal nerve cells were isolated and cultured in F12 complete medium for the first day. Afterwards, the culture medium was changed to NeuM for 2 days and subsequently was replaced by OsM for another day. This one‐day OsM was then collected as the conditional medium for BMSCs in order to imitate the co‐culture of nerve cells and BMSCs. BMSCs (2×10^5^) were co‐cultured indirectly via conditional medium for 48 h before their total RNA was collected using TRIzol Reagent (Invitrogen).

### Eukaryotic Transcriptome Sequencing

After extracting total RNA from the sample, Oligo (dT) magnetic beads enrich eukaryotic mRNA (if prokaryotic, rRNA was removed using a reagent kit to enrich mRNA). Add interruption reagents to break mRNA into short fragments, use the interrupted mRNA as a template, synthesize a single stranded cDNA using six base random primers, then prepare a two stranded synthesis reaction system to synthesize a double stranded cDNA, and purify the double stranded cDNA using a reagent kit. Purified double stranded cDNA was subjected to end repair, A‐tail addition and sequencing adapter connection, followed by fragment size selection and PCR amplification. After passing the quality inspection with Agilent 2100 Bioanalyzer, the constructed library was sequenced using Illumina HiSeqTM4000 or other sequencers. The data obtained from Illumina HiSeqTM2000 sequencing was called raw reads or raw data, followed by quality control (QC) of raw reads to determine whether the sequencing data was suitable for subsequent analysis. After quality control, clean reads were filtered and aligned to the reference sequence using tophat/bowtie2. After comparison, determine whether the alignment results have passed the second quality control (QCof alignment) by analyzing the distribution and coverage of reads on the reference sequence. If passed, a series of subsequent analyses will be conducted, including gene expression, gene structure optimization, variable splicing, new transcript discovery and coding ability prediction, SNP detection, etc.

### ELISA

The serum level of CGRP were evaluated using Mouse CGRP‐I ELISA kit (EIAM‐CGRP‐1) from RayBiotech (Peachtree Corners, GA, USA) according to manufactory's instructions.

### Western Blotting

Western blotting was performed to assess the epigenetic mechanism underlying the effects of CGRP coordinating with microspheres on osteogenesis. The protein expression levels of enhancer of zeste homolog (EZH2) and Tri‐Methyl‐Histone H3 were measured, and GAPDH expression was used as a reference. BMSCs (3×10^5^) were cultured with extraction medium in 6‐well plates for 1 hour. 1 nм recombinant mouse CGRP (ABclonal, RP02196) was for one group and the other group added 1 nм Olcegepant (BIBN‐4096) (MedChemExpress, China). Total protein was extracted with RIPA lysis buffer (EpiZyme, Shanghai, China) supplemented with 1% protease inhibitor (Beyotime, China) at 4 °C for 5 min, and the samples were centrifuged at 12,000 rpm for 15 min. Then, the cellular supernatants were collected, and the protein concentrations were quantified with a BCA protein assay kit (Thermo Fisher, USA) and then dissolved in 5 × SDS‐PAGE loading buffer. Total proteins (20 µg) were electrophoresed in 4–20% SDS‐PAGE gels (Epizyme Biotech, LK102) using a Miniprotein III system (Bio‐Rad, USA) and were transferred to NC membranes (Epizyme Biotech, WJ004S) for 1.5 h, followed by overnight incubation with primary antibody against EZH2 (1:500; Abcam, ab191080), KDM6A (1:1000; Abcam, ab253183), Histone3 (1:1000; Abcam, ab176840), H3K27me3 (1:1000; Cell Signaling Technology, 9733T) and GAPDH (1:2000; Beyotime, AG019) at 4 °C. Then NC membranes were washed three times with TBST solution (Beyotime, P0222) and were incubated at room temperature for 1 h in HRP‐conjugated secondary antibodies (1:5000; Absin, abs20040). The protein bands were visualized, and images were captured with an automated luminescent image analysis system (Tanon, China)

### Statistic Analysis

All quantitative data were expressed as the mean ± standard deviation (SD). One‐way ANOVA, two‐way ANOVA, and t‐tests were performed using GraphPad Prism 9 software (GraphPad SoftwareInc., USA) as statistical analysis. P <0.05 was regarded as statis‐tically significant.

## Conflict of Interest

The authors declare no conflict of interest.

## Author Contributions

G.K. and T.Y. contributed equally to this work. G.K. and T.Y. performed methodology; L.S. and C.S. performed investigation; L.K. performed conceptualization; T.Y. performed revision of this manuscript; Z.M. performed project administration.

## Supporting information

Supporting Information

## Data Availability

The data that support the findings of this study are available from the corresponding author upon reasonable request.
