# Supplementary material for: Sensory Nerve Regulation via H3K27 Demethylation Revealed in Akermanite Composite Microspheres Repairing Maxillofacial Bone Defect
Source: Adv Sci (Weinh). 2024 Jun 14;11(30):2400242. doi: 10.1002/advs.202400242 (PMC11321702; doi:10.1002/advs.202400242)
Supplement: Supplementary file 1 — Supporting Information [file ADVS-11-2400242-s001.pdf]

ADVANCED SCIENCE
Open Access

# Supporting Information

for *Adv. Sci.*, DOI 10.1002/advs.202400242

Sensory Nerve Regulation via H3K27 Demethylation Revealed in Akermanite Composite Microspheres Repairing Maxillofacial Bone Defect

*Kaijun Gu, Yu Tan, Sitong Li, Siyue Chen, Kaili Lin*, Yanmei Tang* and Min Zhu**

# Supporting Information

**Title: Sensory Nerve Regulation via H3K27 Demethylation Revealed in Akermanite Composite Microspheres Repairing Maxillofacial Bone Defect**

***Authors:*** *Kaijun Gu[1,#], Yu Tan[2,#], Sitong Li[1], Siyue Chen[1], Kaili Lin[1,*], Yanmei Tang[1,*], Min Zhu[1,*]*

*(#:co-first authors, *: co-corresponding authors)*

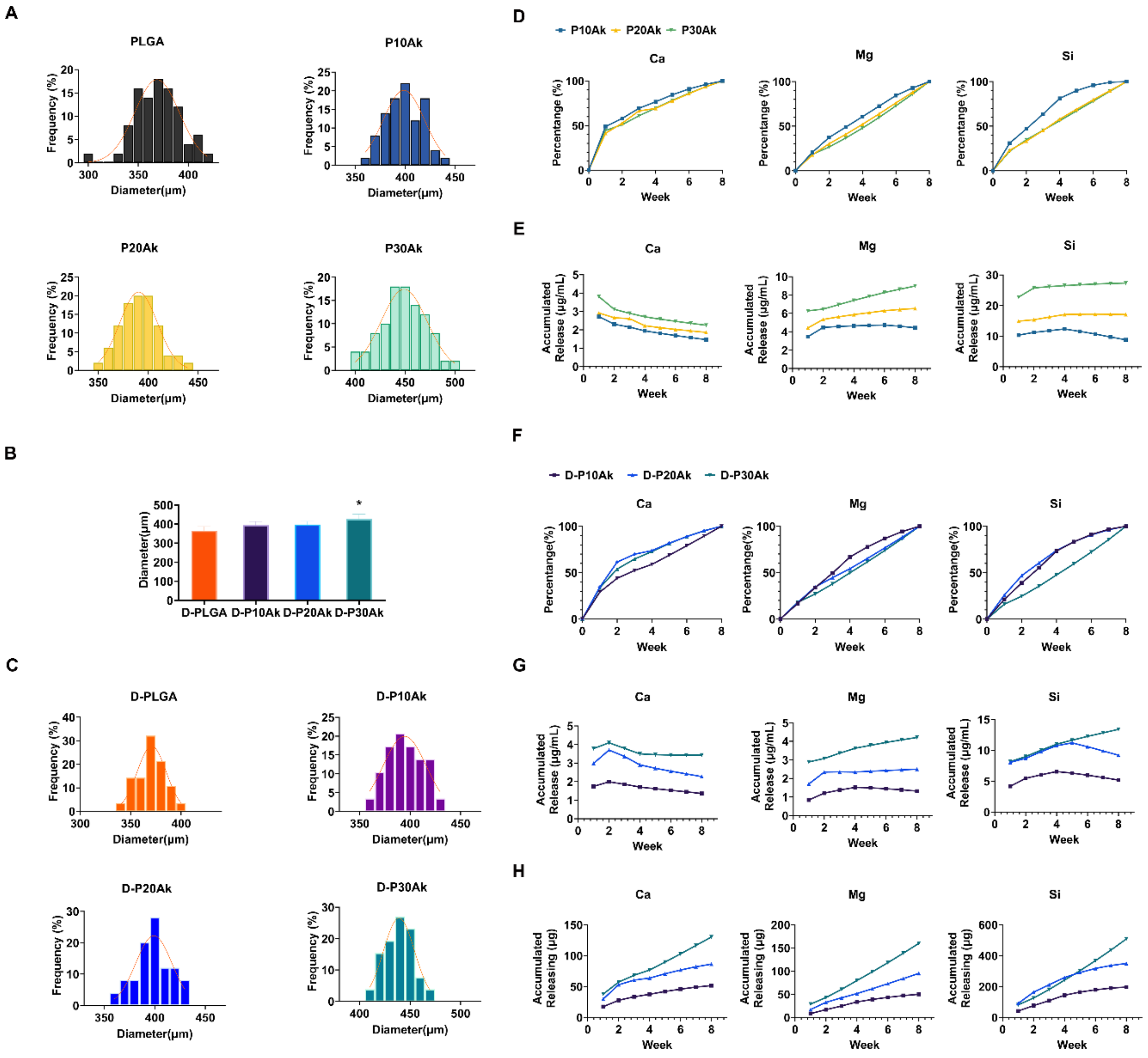


**Figure S1. Supplementary for the characterization of the microspheres**

A. The diameter distribution map of P-Ak composite microspheres.

B. Average size of 4 groups of polydopamine-coated composite microspheres.

C. The diameter distribution map of D-P-Ak composite microspheres. * P<0.05

D. The ion release percentage curve of P-Ak composite microspheres in 8 weeks. Every 30mg microspheres were immersed in 10mL PBS at 37℃. 4mL of the fluid was refreshed every week.
E. The accumulated release concentration curve of P-Ak composite microspheres in 8 weeks. The 4mL extraction taken out every week was calculated as well.
F. The ion release percentage curve of D-P-Ak composite microspheres in 8 weeks.
G. The accumulated release concentration curve of D-P-Ak composite microspheres in 8 weeks.
H. Accumulative ion release profiles of D-P-Ak composite microspheres in 8 weeks.

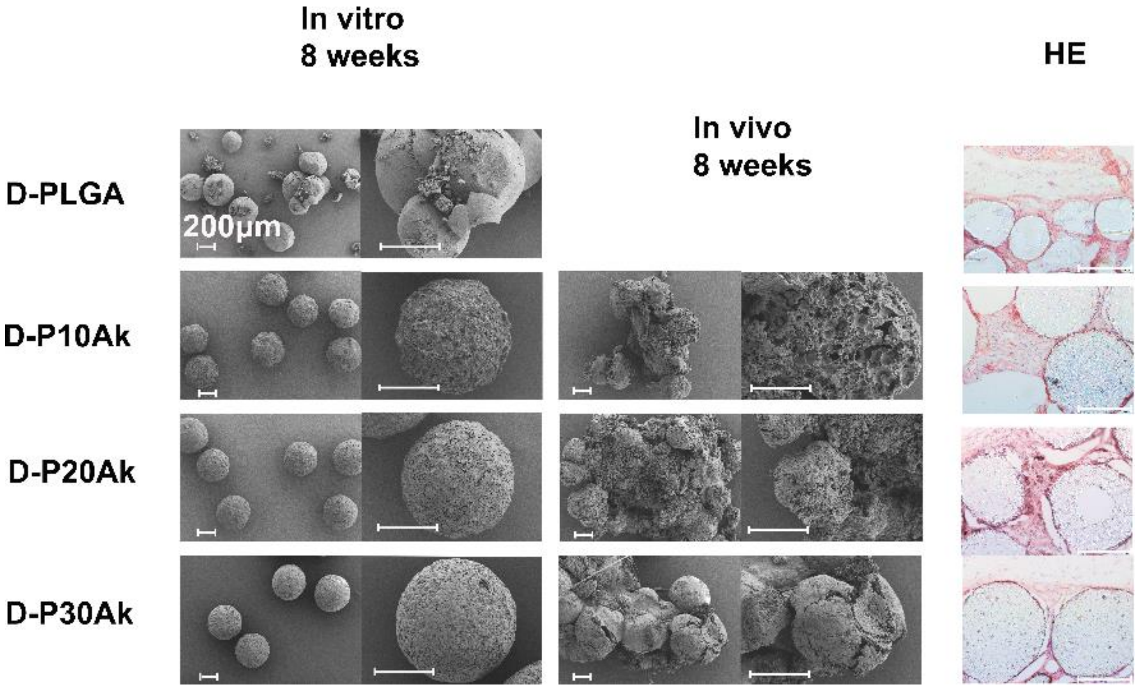


**Figure S2. Biodegradation of D-P-Ak microspheres in vitro and in vivo in 8 weeks**

Morphology of degraded D-PLGA-Ak microspheres in vivo were observed with SEM. From Figure S2, D-PLGA-Ak microspheres had lost their original shape and agglomerated into bulks after 8 weeks of implantation subcutaneously. The corrosion observed in Figure S2 resulted in a rougher surface on the degraded microspheres. D-PLGA microspheres demonstrated a faster degradation rate than others, and collapses on the surface were detected after 8 weeks. The remaining microspheres along with surrounding adherent tissues were harvested and an instant frozen section was performed. H&E staining were applied to detect inflammation around the implanted microspheres. Fig S2 showed normal connective tissue growth around the microspheres, no aggregation of inflammatory cells and no clustered necrotic cells or cell fragments observed. Scale bar is 200 μm.

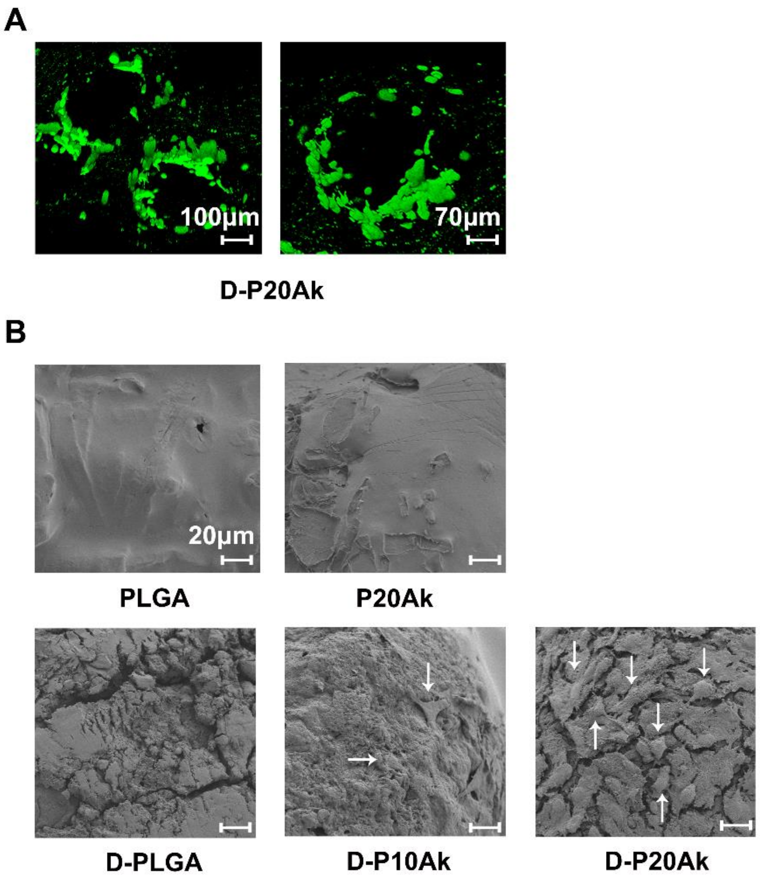


**Figure S3. Supplementary images of cell adhesion and spreading on the surface of the microspheres.**

A. The 3D image of D-P20Ak with BMSCs adhesion taken by confocal microscope after 24h incubation. Scale bar is 100 μm.

B. Biological scanning electron microscopy imaging of BMSCs adhesion and spreading on both polydopamine-coated and uncoated composite microspheres after 24h incubation. Arrows point at the adhered cells. Scale bar is 20 μm.

The surface of the P-Ak composite microspheres without dopamine coating was relatively smooth. The D-PLGA microspheres had a rough surface, but cell colonization was still rare. Individual cell adhesion and colonization could be observed on the surface of D-P10Ak microspheres. On the surface of D-P20Ak microspheres, clusters of cells adhered and spread out, with a larger spreading area and wider contact with the microsphere. Good cell adhesion is an important prerequisite for subsequent proliferation and differentiation.

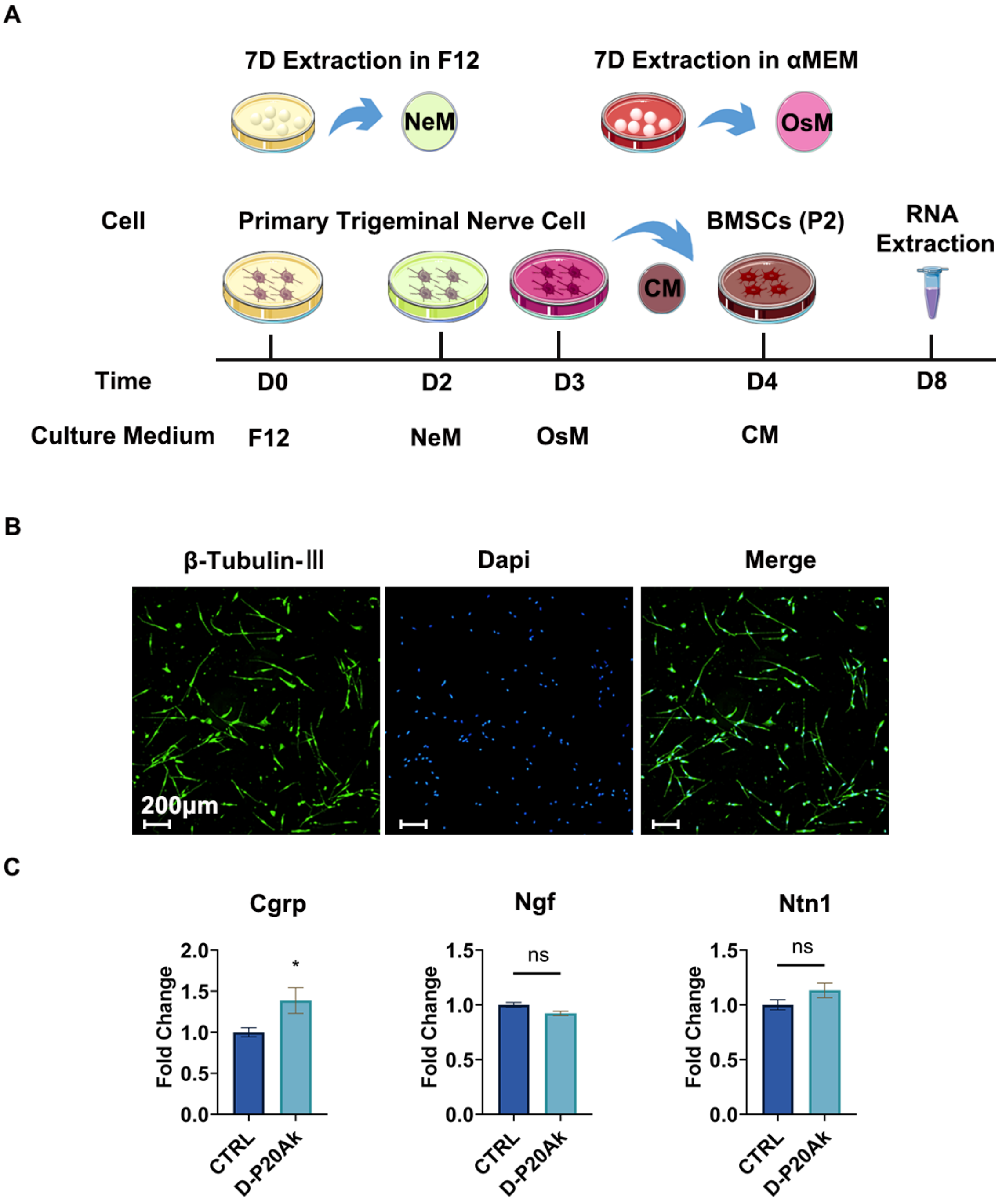


**Figure S4. In vitro indirect co-culture using isolated trigeminal nerve cells.**

A. The protocol of indirect co-culture of microspheres, trigeminal sensory nerve cells and BMSCs in vitro. Outcomes were displayed in Figure 5D.

B. Immunofluorescence of isolated and cultured trigeminal cells in vitro at day 3. β-tubulin-III is a marker for sensory neurons. Scale bar is 200μm.

C. mRNA expression of trigeminal nerve cells cultured with NeM for 4 days. CTRL was trigeminal nerve cells cultured with complete F12 medium. CGRP was the most significant marker of differential expression in the coding genes of neural related proteins. 3 duplicates were tested for each group. * $P<0.05$.

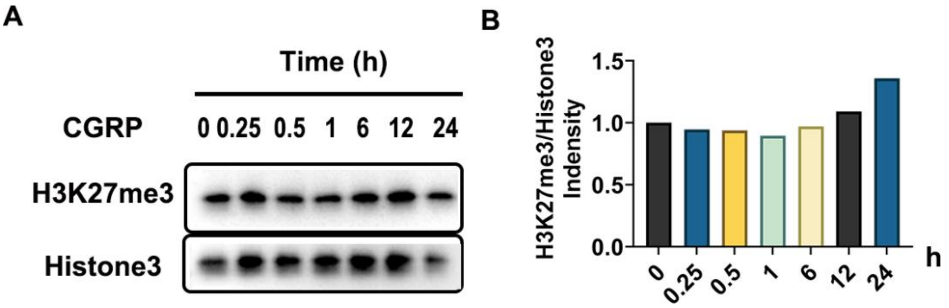


**Figure S5. The methylation level of H3K27me3 treated with CGRP at each point of time within 24 hours.**

A. Western blotting of BMSCs lysis with certain length of 1nM CGRP treatment. CTRL, BMSCs cultured with only complete α-MEM.
B. Quantitative analysis of strip intensity of every group. 1-hour-treatment group shows the most reduction on H3K27 trimethylation. All results are normalized to the expression of histone3.

**Table S1.** Primers used for rat targeted genes

| Genes | Primer (F=Forward, R=Reverse) |
|---|---|
| ALP-F | AACGTGGCCAAGAACATCATCA |
| ALP-R | TGTCCATCTCCAGCCGTGTC |
| Runx2-F | CACAAGTGCGGTGCAAACTT |
| Runx2- R | AAGAGGCTGTTTGACGCCAT |
| OCN-F | AATAGACTCCGGCGCTACCT |
| OCN-R | TAGATGCGCTTGTAGGCGTC |
| OPN-F | AGACTGGCAGTGGTTTGCTT |
| OPN-R | AGTGTTTGCTGTAATGCGCC |
| GAPDH-F | GGCACAGTCAAGGCTGAGAATG |
| GAPDH-R | ATGGTGGTGAAGACGCCAGTA |

**Table S2.** Primers used for mouse targeted genes

| Gene | Primer (F=Forward, R=Reverse) |
|---|---|
| cgrp-F | TGAGGGCTCTAGTGTCACTGCT |
| cgrp-R | GTAACCTTCATTCTGGGGCTGT |

| | |
|---|---|
| alp-F | TTGCCAAGCTGGGAAGAACA |
| alp-R | ACCCCGCTATTCCAAACAGG |
| runx2-F | CCTCTGACTTCTGCCTCTGG |
| runx2-R | TATGGAGTGCTGCTGGTCTG |
| β-actin-F | TTGCTGACAGGATGCAGAAG |
| β-actin-R | AAGGGTGTAAAACGGAGCTC |
| kdm6a-F | GGCTACTGGGGTGTTTTGAA |
| kdm6a-R | TCCAGGTCGCTGAATAAACC |
| kdm6b-R | CCCCCATTTCAGCTGACTAA |
| kdm6b-F | CTGGACCAAGGGGTGTGTT |
| ezh2-F | GGGAGAGAACAACGATAAAGAAGAAGA |
| ezh2-R | GGCTTCATCTTTATTGGTGTTTGACA |
| gapdh-F | AAACCCATCACCATCTTCCA |
| gapdh-R | GTGGTTCACACCCATCACAA |
| osx-F | GATGGCGTCCTCTCTGCTTG |
| osx-R | TCTTTGTGCCTCCTTTCCCC |
| ocn-F | CCCTGAGTCTGACAAAGCCT |
| ocn-R | GCGGTCTTCAAGCCATACTG |
| opn-F | GGAAACCAGCCAAGGTAAGC |
| opn-R | TGCCAATCTCATGGTCGTAG |
| ngf-F | GACCACAGCCACAGACATCAAGG |
| ngf-R | GGCACCCACTCTCAACAGGATTG |
| ntn1-F | GCCTTCCTCACCGACCTCAATAAC |
| ntn1-R | CTTCTTGCCGAGCGACAGAGTG |
